# Unveiling clinicopathologic features and outcomes for endoscopic submucosal dissection of early gastric cancer at gastric angulus in China

**DOI:** 10.1186/s12885-024-12610-1

**Published:** 2024-07-30

**Authors:** Qiaoyan Wu, Tongyu Li, Yangyang Cui, Haizhong Jiang, Yangbo Fu, Qi Jiang, Xiaoyun Ding

**Affiliations:** 1 Department of Gastroenterology, The First Affiliated Hospital of Ningbo University, No. 59, Liuting Street, Ningbo, Zhejiang Province 315010 China; 2 Ningbo Key Laboratory of Translational Medicine Research on Gastroenterology and Hepatology, No. 59, Liuting Street, Ningbo, Zhejiang Province 315010 China; 3 Department of Hematology, The First Affiliated Hospital of Ningbo University, No. 59, Liuting Street, Ningbo, Zhejiang Province 315010 China; 4 Department of Histopathology, Ningbo Diagnostic Pathology Center, No. 685 North Huancheng Road, Ningbo, Zhejiang Province 315021 China

**Keywords:** Early gastric cancer, Endoscopic submucosal dissection, Location, Gastric angulus, Submucosal fibrosis

## Abstract

**Background:**

With advances in endoscopic submucosal dissection (ESD) technique, an increasing number of the Chinese population are being diagnosed with early gastric cancers (EGCs) at gastric angulus. However, the relationship between gastric angulus and EGCs remains obscure.

**Objectives:**

We aimed to unveil the unreported location characteristics of gastric angulus in Chinese EGC patients and the correlation between the degree of submucosal fibrosis and ESD outcomes.

**Methods:**

We retrospectively reviewed the medical records of EGC patients treated with ESD from January 2010 to March 2023. We retrospectively investigated and analyzed 740 EGC patients using multiple analyses.

**Results:**

Following gastric antrum (53.1%), the gastric angulus (21.8%) emerged as the second-most prevalent site for EGCs. It had highest incidence of severe submucosal fibrosis and ulceration than the other parts. Multivariate analysis showed independent associations of submucosal fibrosis at the angulus with ulceration (OR: 3.714, 95% CI: 1.041–13.249), procedure duration (OR: 1.037, 95% CI: 1.014–1.061), and perforation complication (OR: 14.611, 95% CI: 1.626-131.277) (all *P* < 0.05).

**Conclusions:**

The gastric angulus demonstrates the highest incidence of severe submucosal fibrosis and ulceration for EGCs identified by ESD. This condition is linked to unfavorable outcomes, typically increased perforation risks and prolonged operation duration. Therefore, meticulous dissection is crucial for patients with EGCs in the gastric angulus.

**Supplementary Information:**

The online version contains supplementary material available at 10.1186/s12885-024-12610-1.

## Key Summary

**Established knowledge on this study:**

- Advances in endoscopic technology have led to higher EGC detection and survival improvements.

- The specific relationship between the gastric angulus and EGCs was not clearly understood.

- The role of gastric angulus in EGC occurrence and its treatment outcomes were not extensively explored.

**Significance of this study:**

- Gastric angulus identified as a second-most common EGC site with high submucosal fibrosis and ulceration.

- Submucosal fibrosis at angulus related to various risk factors and complications like longer surgeries and hospitalization.

- The high prevalence of fibrosis risks underscores the need for meticulous dissection in EGC cases at the gastric angulus.

**Supplementary Information:**

The online version contains supplementary material available at 10.1186/s12885-024-12610-1.

## Introduction

Gastric cancer is a prevalent malignancy worldwide. Eastern Asia, particularly China, has a greater prevalence of gastric cancer and related death than other regions [1]. The stage of gastric cancer is highly associated with morbidity and mortality, and invasion degree, the number of metastatic lymph nodes, and distant metastasis are critical determinants for the treatment modality [2]. Irrespective of lymph node metastases, early gastric cancer (EGC) involves tumor invasion of the mucosa or submucosa [3]. In recent years, advances in population-based screening and endoscopic technologies have substantially increased the diagnosis rates of EGC in China, resulting in decreased mortality and a higher 5-year survival rate [4, 5].

Endoscopic submucosal dissection (ESD) has emerged as the predominant modality for the dissection of EGC [6]. The utilization of ESD has exhibited a multitude of benefits in comparison to conventional Endoscopic Mucosal Resection (EMR), including decreased expenses, minimized physical trauma, expedited healing, and improved overall quality of life [7]. The success rate of ESD is contingent upon the specific site and presence of submucosal fibrosis, as well as the proficiency of the endoscopist and the overall state of a tumor [8]. If sites of EGC with different features can be determined, a more accurate and individualized management strategy for those patients will be realized. Chung and colleagues, along with the prevailing research, partitioned the stomach into three equidistant segments: the upper, middle, and lower regions, as a means to differentiate the location of the EGC lesion to facilitate tailored management [9]. However, to date, no published research has focused on the clinicopathological relevance of EGC at the gastric angulus, a tiny concave site of the stomach where EGCs are frequently detected in Chinese patients. Notably, the protruding anatomical features, coupled with factors such as paradoxical movement, abundant vasculature, render ESD exceptionally arduous in this region [10].

Submucosal fibrosis, typically arising from inflammation or the invasion of tumors, presents a particular challenge in effectively separating the tumor tissue from the muscular layer, especially at the angulus [11]. The extent of submucosal fibrosis consequently prolongs the procedure duration and increases the likelihood of complications such as perforations, thereby diminishing the efficacy of ESD. Furthermore, Jae Yoon Jeong e t al. further elucidated that the midsection of the stomach showed a heightened occurrence of endoscopic submucosal fibrosis, establishing itself as a risk factor for this condition [12]. According to Chinese division, the middle part primarily comprises the gastric body, while the gastric angulus is located in the lower region, with the extent of submucosal fibrosis remains undetermined [13, 14]. Regrettably, the interplay among the gastric angulus, degree of submucosal fibrosis, and the outcomes of ESD in EGC has remained largely overlooked or understudied.

Accordingly, the objectives of this study were twofold. Firstly, to examine the incidence of EGC treatable by ESD at the gastric angulus. Secondly, to compare the various clinicopathological characteristics of EGC at the gastric angulus to facilitate accurate clinical management and boost success rate.

## Materials and methods

### Patients

This study involved a retrospective analysis of medical records from 820 consecutive patients diagnosed with EGCs who underwent gastric ESD at our institution in Ningbo City, Zhejiang Province, China, among the Han population. The Ethics Committee of our institution approved the research (2023-122RS-01). The data collection spanned from January 2010 to March 2023. Our analysis was restricted to instances that satisfied the ESD criteria stipulated in the Japanese Gastric Cancer Treatment Guidelines [15].

The criteria for including patients were as follows: (1) Patients confirmed to have EGC through gastroscopy and biopsy; (2) no contraindications such as distant metastases were evident from relevant imaging studies, the patient was eligible for ESD, and informed consent was obtained prior to the procedure. The criteria for excluding patients included: (1) Patients with gastric mucosal tumors who did not undergo ESD; (2) patients with incomplete follow-up data; and (3) patients suffering from severe cardiovascular, hematological, neuropsychiatric disorders, or significant liver and kidney impairment, among other similar conditions [16].

**Fig. 1:**
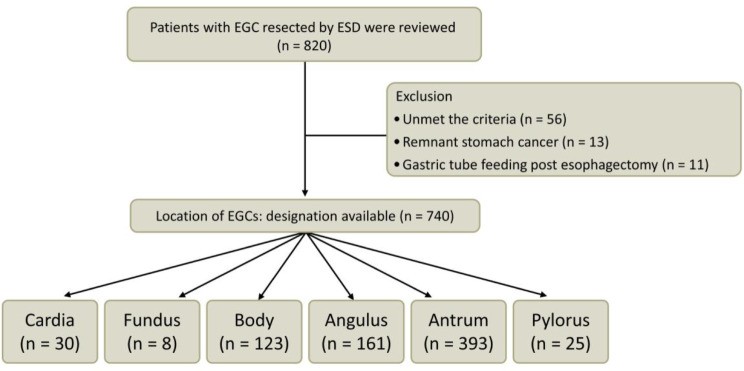
A flowchart shows the study design

Of the total patients, 80 cases were excluded. The reasons for exclusion were as follows: a total of 56 patients failed to satisfy the pre-established criteria, 13 patients had lesions that originated in a remnant stomach, and 11 had undergone a gastric tube placement procedure following esophagectomy. Finally, the present study involved the analysis of 740 patients with EGC located in various regions of the stomach (Figs. 1 and S1). The adequacy of our sample size was substantiated by prior research [9, 17]. The medical records were retrospectively analyzed.

### ESD technique

The initial detection and subsequent lateral resection of EGCs were accomplished through chromoendoscopy and white-light endoscopy with narrow-band imaging, indigo-carmine solution, magnifying endoscopy, or a hybrid approach incorporating these techniques. The ESD procedure was performed by seasoned GI endoscopists, with over a decade of dedication to ESD, adhering faithfully to a standardized methodology. The endoscopists employed a conventional endoscope with a solitary accessory channel, specifically the GIF-Q260J model, manufactured by Olympus Optical Co, Ltd.

### Classification of submucosal fibrosis during ESD

The previous categorization system was used to determine the degree of submucosal fibrosis [18]. The present system has been formulated based on the observations made while administering a saline solution containing methylthionine chloride. The classification system employed in this study involved three distinct stages of fibrosis (Figure S2).

### Histopathological evaluation

Submucosal fibrosis was further corroborated through histopathological evaluation using hematoxylin and eosin and Masson’s trichrome staining. The degree of submucosal fibrosis was jointly determined by two experienced pathologists, who were blinded to any clinical information. Depending on the severity, histologic submucosal fibrosis was graded as 0, 1, or 2. A score of 0 indicated no fibrosis, 1 indicated mild fibrosis and 2 indicated severe fibrosis (Figure S3).

### Statistical analysis

SPSS (26.0) was used for all data analysis. Continuous variables were reported as mean ± standard deviation in normal distribution or median (interquartile range) in the non-normal distribution, while categorical variables were presented as numbers (percentage). Comparisons between continuous variables were performed using the independent sample Student’s t-test or Wilcoxon Mann–Whitney test or one-way analysis of variance (ANOVA). Categorical variables were compared using the Pearson chi-square test and Fisher’s exact test. The Bonferroni correction was applied post-hoc to adjust the significance level for multiple comparisons. A univariate logistic regression was carried out to evaluate factors related to severe submucosal fibrosis at the gastric angulus. Factors shown to be significant were entered into multivariate logistic regression models to identify those independently predictive of F2 fibrosis at the stomach angulus. Cohen’s conventions for interpretation of η^2^ values suggest that effect sizes of about 0.01 are small, 0.06 to 0.14 are medium. The groups were further evaluated with Cramer V/ Phi statistic to assess effect sizes for the Pearson chi-square test and Fisher’s exact test. A magnitude of 0.1 was considered small, 0.3 medium, and 0.5 large. Statistical significance was defined as *P* < 0.05.

## Results

### Demographics of EGC patients

Our analysis comprised 740 patients with EGC who underwent ESD. The study population had a mean age of 64.4 ± 8.1 years, with a majority of 488 (65.9%) patients being male. The primary sites of occurrence for EGCs were observed to be the antrum and the angulus, accounting for 53.1% and 21.8% of cases, respectively. The predominant macroscopic appearance type was flat or depressed in 461 (62.3%) patients. Through dual confirmation *via* endoscopy and histopathological evaluation, 49 patients, constituting 6.6% of the sample, were diagnosed with severe submucosal fibrosis. The percentage of *en bloc* resection was 97.7%. The most common complication related to the procedure was delayed bleeding, which was observed in only 23 (3.1%) cases. In two instances, the bleeding was uncontrollable via endoscopy, resulting in the need for emergency surgery and a blood transfusion.

Additional radical surgical interventions were performed in 27 (69.2%) of the 39 noncurative resections; however, of these 27 patients, 3 had metastatic lymph nodes. The remaining 12 patients rejected surgery due to unwillingness, comorbidities, or advanced age (Table 1).

**Table 1:** Baseline characteristics of EGC patients

Characteristic	Total (*n* = 740)
Male, n (%)	488 (65.9)
Age, yr	64.4 (8.1)
< 65, n (%)	393 (53.1)
≥ 65, n (%)	347 (46.9)
Comorbidities, n (%)	
COPD	22 (3.0)
Diabetes	65 (8.8)
Hypertension	254 (34.3)
Location, n (%)	
Cardia	30 (4.1)
Fundus	8 (1.1)
Body	123 (16.6)
Angulus	161 (21.8)
Antrum	393 (53.1)
Pylorus	25 (3.4)
Tumor size (mm)	
< 20 mm, n (%)	522 (70.5)
≥ 20 mm, n (%)	218 (29.5)
Macroscopic type, n (%)	
Elevated	279 (37.7)
Flat or depressed	461 (62.3)
Submucosal fibrosis, n (%)	
No or mild (F0 + F1)	691 (93.4)
Severe (F2)	49 (6.6)
Ulceration, n (%)	155 (20.9)
Number of tumors, n (%)	
Single	652 (88.1)
Multiple	88 (11.9)
Depth of invasion, n (%)	
Mucosal lesion	696 (94.1)
Submucosal invasion	44 (5.9)
Resection margin involvement, n (%)	
Lateral margin (+)	9 (1.2)
Vertical margin (+)	10 (1.4)
Lymphatic invasion, n (%)	11 (1.5)
Histology, n (%)	
Differentiated	681 (92.0)
Undifferentiated	59 (8.0)
*En bloc* resection, n (%)	723 (97.7)
Complete resection, n (%)	711 (96.1)
Curative resection, n (%)	701 (94.7)
Complications, n (%)	
Perforation	13 (1.8)
Delayed bleeding	23 (3.1)
Additional gastrectomy, n (%)	27 (3.6)
Lymph node metastasis, n (%)	3 (0.4)
Procedure time (min)	80.0 (30.0)
Hospital stay (day)	9.0 (2.0)

### Comparison between the angulus and non-angulus parts of the stomach: univariate and multivariate analyses

The clinicopathological features of patients with EGC at different locations were compared. While being the smallest part, the gastric angulus exhibited the highest occurrence of severe submucosal fibrosis and ulceration among patients with EGC (*P* < 0.001). The findings also suggested that gastric angulus exhibited the largest proportion of flat or depressed-type lesions (69.6%), lymphatic invasion (3.1%), undifferentiated histology (10.6%), and complications of delayed bleeding (5.0%). However, the differences were negligible (*P* > 0.05). Additionally, due to the mucosa dissection had to reach deeply into the gastric muscularis during ESD, 11 cases at the gastric angulus experienced micro-perforations that were immediately closed with endoscopic clips, whereas 2 cases at the gastric body had micro-perforations that were surgically repaired with omentum patches (Table 2).

**Table 2:** Clinicopathology of patients among the different EGC locations

Variables	Cardia + Fundus* (*n* = 38)	Body (*n* = 123)	Angulus (*n* = 161)	Antrum + Pylorus* (*n* = 418)	*P* valve	η2/Cramer V
Male sex, n (%)	28 (73.7)	91 (74.0)	109 (67.7)	260 (62.2)	0.061	0.100
Age, yr	62.7 (8.0)	63.4 (7.4)	65.3 (9.0)	64.6 (8.0)	0.511	0.008
< 65, n (%)	25 (65.8)	71 (57.7)	80 (49.7)	217 (51.9)	0.212	0.078
≥ 65, n (%)	13 (34.2)	52 (42.3)	81 (50.3)	201 (48.7)		
Comorbidities, n (%)						
COPD	2 (5.3)	7 (5.7)	3 (1.9)	10 (2.4)	0.140	0.082
Diabetes	5 (13.2)	11 (8.9)	7 (4.3)	42 (10.0)	0.092	0.088
Hypertension	11 (28.9)	44 (35.8)	45 (28.0)	154 (36.8)	0.197	0.080
Tumor size (mm)					< 0.001	0.380
< 20 mm, n (%)	28 (73.7)	74 (60.2)	97 (60.2)	323 (77.3)		
≥ 20 mm, n (%)	10 (26.3)	49 (39.8)	64 (39.8)	95 (22.7)		
Macroscopic type, n (%)					0.112	0.090
Elevated	18 (47.4)	51 (41.5)	49 (30.4)	161 (38.5)		
Flat or depressed	20 (52.6)	72 (58.5)	112 (69.6)	257 (61.5)		
Submucosal fibrosis, n (%)					< 0.001	0.620
No or mild (F0 + F1)	36 (94.7)	116 (94.3)	131 (81.4)	408 (97.6)		
Severe (F2)	2 (5.3)	7 (5.7)	30 (18.6)^#^	10 (2.4)		
Ulceration, n (%)	4 (10.5)	22 (17.5)	61 (37.9)^#^	68 (16.3)	< 0.001	0.522
Number of tumors, n (%)					0.301	0.071
Single	35 (92.1)	103 (83.7)	140 (87.1)	374 (89.5)		
Multiple	3 (7.9)	20 (16.4)	21 (12.9)	44 (10.5)		
Depth of invasion, n (%)					0.063	0.105
Mucosal lesion	34 (89.5)	110 (89.4)	152 (94.4)	400 (95.7)		
Submucosal invasion	4 (10.5)	13 (10.6)	9 (5.6)	18 (4.3)		
Resection margin involvement, n (%)						
Lateral margin (+)	2 (5.3)	0 (0.0)	3 (1.9)	4 (1.0)	0.086	0.101
Vertical margin (+)	1 (2.6)	2 (1.6)	3 (1.9)	4 (1.0)	0.739	0.043
Lymphatic invasion, n (%)	0 (0.0)	2 (1.6)	5 (3.1)	4 (1.0)	0.234	0.076
Histology, n (%)					0.501	0.058
Differentiated	34 (89.5)	114 (92.7)	144 (89.4)	389 (93.1)		
Undifferentiated	4 (10.5)	9 (7.3)	17 (10.6)	29 (6.9)		
*En bloc* resection, n (%)	38 (100.0)	121 (98.4)	149 (92.5)	415 (99.3)	< 0.001	0.183
Complete resection, n (%)	37 (97.4)	119 (96.7)	146 (90.7)	409 (97.8)	0.004	0.148
Curative resection, n (%)	36 (94.7)	118 (95.9)	144 (89.4)	403 (96.4)	0.018	0.216
Complications, n (%)						
Perforation	0 (0.0)	2 (1.6)	11 (6.8)	0 (0.0)	< 0.001	0.209
Delayed bleeding	1 (2.6)	4 (3.3)	8 (5.0)	10 (2.4)	0.497	0.059
Additional gastrectomy, n (%)	2 (5.3)	5 (4.1)	7 (4.3)	13 (3.1)	0.830	0.035
Lymph node metastasis, n (%)	0 (0.0)	2 (1.6)	1 (0.6)	0 (0.0)	0.092	0.094
Procedure time (min)	75.0 (60.0)	75.0 (50.0)	83.0 (40.0)	70.0 (30.0)	0.021	0.038
Hospital stay (day)	11.0 (3.1)	9.0 (3.0)	9.0 (2.0)	9.0 (2.0)	0.515	0.031

*Due to limited cases and anatomical proximity, the groups for gastric cardia and fundus were combined, as were the groups for pylorus and antrum, for further comparison in the table. ^#^The gastric angulus exhibited a significantly higher incidence of severe submucosal fibrosis and ulceration than the rest parts (*P* < 0.001)

Prior studies indicated a prevalence of endoscopic submucosal fibrosis in the gastric body, with the gastric angulus undetermined. Thus, we further performed the univariate analysis of the patients treated with ESD at the gastric angulus or body. The results unveiled significantly elevated rates of severe submucosal fibrosis, ulceration, and perforation complications, extended hospitalization, and lower percentages of *en bloc* resection, complete resection, and curative resection at the gastric angulus compared to the gastric body (*P* < 0.05; Table S1). The detailed comparison of severe submucosal fibrosis and ulceration in the gastric body and angulus further revealed that differences mainly occurred along the lesser and greater curvature sides (Table S2 and Figure S4).

### Clinicopathologic factors and outcomes relating to severe submucosal fibrosis at the gastric angulus

Subsequently, we conducted both univariate and multivariate analyses on patients treated with ESD who had EGC at the gastric angulus. Univariate analysis revealed that, a bivariate correlation was observed between male gender, tumor size, vertical margin involvement, and the extent of submucosal fibrosis. Compared to their non-severe counterparts, cases of severe submucosal fibrosis were also associated with higher incidences of submucosal invasion, ulceration, undifferentiated histology, perforation complications, and the need for additional gastrectomy, while exhibiting lower rates of en bloc resection, complete resection, and curative resection, all of which are indicative of unfavorable outcomes in cases of EGC at the gastric angulus (*P* < 0.05; Table 3). Additionally, the F2 group had extended procedure duration than the F0 + F1 group (*P* = 0.004). Likewise, the F2 group had longer hospital stay than the F0 + F1 group (*P* = 0.027). However, submucosal fibrosis seems to have little effect on delayed bleeding (*P* > 0.05). Multivariate logistic regression analysis showed endoscopic ulceration, procedure duration, and perforation complication to be independent factors predictive of F2 fibrosis (Table 4).

**Table 3:** Characteristics and outcomes relating to severe submucosal fibrosis: a univariate analysis

Variables	F0 + F1 (*n* = 131)	F2 (*n* = 30)	*P* valve	Cohen’s/Phi
Male sex, n (%)	84 (64.1)	25 (83.3)	0.042	0.360
Age, yr	64.4 (9.1)	70.3 (6.7)	0.061	0.043
< 65, n (%)	62 (47.3)	18 (60.0)	0.099	0.099
≥ 65, n (%)	69 (52.7)	12 (40.0)		
Comorbidities, n (%)				
COPD	2 (1.5)	1 (3.3)	0.052	0.052
Diabetes	5 (3.8)	2 (6.7)	0.054	0.054
Hypertension	36 (27.5)	9 (30.0)	0.823	0.022
Tumor size (mm)			< 0.001	0.623
< 20 mm, n (%)	87 (66.4)	10 (33.3)		
≥ 20 mm, n (%)	44 (33.6)	20 (66.7)		
Macroscopic type, n (%)			0.207	0.099
Elevated	37 (28.2)	12 (40.0)		
Flat or depressed	94 (71.8)	18 (60.0)		
Depth of invasion, n (%)			0.001	0.359
Mucosal lesion	128 (97.7)	24 (80.0)		
Submucosal invasion	3 (2.3)	6 (20.0)		
Ulceration	37 (28.2)	24 (80.0)	< 0.001	0.541
Number of tumors, n (%)			0.396	0.091
Single	112 (85.5)	28 (93.3)		
Multiple	19 (14.5)	2 (6.7)		
Resection margin involvement, n (%)				
Lateral margin (+)	2 (1.5)	1 (3.3)	1.000	0.052
Vertical margin (+)	0 (0.0)	3 (10.0)	0.006	0.288
Lymphatic invasion, n (%)	4 (3.1)	1 (3.3)	1.000	0.006
Histology, n (%)			0.004	0.251
Differentiated	122 (93.1)	22 (73.3)		
Undifferentiated	9 (6.9)	8 (26.7)		
*En bloc* resection, n (%)	125 (93.1)	24(80.0)	0.012	0.392
Complete resection, n (%)	123 (93.9)	23 (76.7)	0.010	0.321
Curative resection, n (%)	122 (93.1)	22 (73.3)	0.004	0.521
Complications, n (%)				
Perforation	2 (1.5)	9 (30.0)	< 0.001	0.439
Delayed bleeding	5 (3.8)	3 (10.0)	0.153	0.112
Additional gastrectomy, n (%)	3 (2.4)	4 (13.3)	0.029	0.311
Lymph node metastasis, n (%)	0 (0.0)	1 (3.3)	0.186	0.165
Procedure time (min)	65 (30.0)	120 (67.5)	0.004	0.112
Hospital stay (day)	9 (2.0)	10.5 (4.8)	0.027	0.067

**Table 4:** Characteristics and outcomes relating to severe submucosal fibrosis: a multivariate analysis

Variables	Odds ratio (95% CI)	*P* valve
Male	2.939 (0.795–10.873)	0.106
Tumor size ≥ 20 mm	2.299 (0.659–8.017)	0.191
Submucosal invasion	5.522 (0.716–42.605)	0.101
Ulceration	3.714 (1.041–13.249)	0.043
Undifferentiated histology	0.768 (0.075–7.811)	0.823
Perforation complication	14.611 (1.626-131.277)	0.017
Additional gastrectomy	0.786 (0.040-15.574)	0.874
Procedure time	1.037 (1.014–1.061)	0.002
Hospital stay	1.051 (0.802–1.378)	0.718

### Analysis of risk factor for severe submucosal fibrosis in different subgroup

We next conducted detailed subgroup analyses based on relevant clinicopathological factors such as histological subtypes and depth of invasion to explore their potential interactions with submucosal fibrosis and ESD outcomes. The results showed that in the subgroup of patients with mucosal lesion invasion, multivariate analysis provided strong evidence that both perforation (OR: 18.475, 95% CI 2.055-166.109, *P* = 0.009) and procedure time (OR: 1.034, 95% CI: 1.010–1.059, *P* = 0.006) were significantly linked to a higher risk of severe submucosal fibrosis (Table S3). Additionally, among patients with differentiated histology, multivariate analysis indicated that both perforation (OR: 11.410, 95% CI: 1.133-114.924, *P* = 0.039) and procedure time (OR: 1.029, 95% CI: 1.004–1.055, *P* = 0.022) were associated with an elevated risk of severe submucosal fibrosis. Conversely, for patients with submucosal invasion, the evidence was insufficient to suggest an association between perforation and procedure time with an increased risk of severe submucosal fibrosis (Table S3). Other clinicopathological factors were not analyzed in subgroups due to the small number of positive cases.

### Comparative long-term follow-up outcomes of angulus EGC

Finally, we conducted a long-term follow-up study on 161 patients with EGC at the gastric angulus. Due to the extended duration of the study, 21 patients were lost to follow-up, leaving 140 patients for analysis. The follow-up data were analyzed using the Kaplan-Meier estimator and log-rank test. During a median follow-up period of 68 months (range 13–101), the disease-free survival (DFS) rate for the F0 + F1 group was 89.6% (104/111), while for the F2 group it was 92.9% (28/29), with no statistically significant difference (*P* = 0.698). Similarly, the overall survival (OS) rate for the F0 + F1 group was 91.3% (105/111), compared to 62.2% (27/29) for the F2 group, also with no statistically significant difference (*P* = 0.577; Figure S5).

## Discussion

Given the increasing necessity of health examinations and the rising expense of medical insurance, EGC detection should be adopted. Multiple studies have suggested approaches to effectively improve the accuracy and rapidity of EGC detection [19–21]. According to a multicenter study, endoscopically detected EGC lesions in the lower, middle, and upper stomachs of 952 Korean patients were 72%, 21%, and 7%, respectively [9]. Consistent with this, Kang et al. further indicated that EGC was most commonly found in the lesser curvature (43.9%) of the stomach [22]. However, no study has yet described EGC at the stomach angulus in patients post-ESD, which is a challenging location for endoscopic maneuverability due to its protruding anatomical structure and other factors. The most pertinent report was a multicenter Spanish study involving 225 ESD patients that identified the incisura, part of the middle stomach, as the third most common site for EGC and suggested a potential link between ESD difficulty and lesion location, including the incisura [23]. We thus examined the endoscopic findings of 740 patients following ESD, discerning that the gastric angulus, a small bend between the stomach body and antrum, constituted the second most common site for detecting EGCs.

We additionally conducted a more in-depth investigation of the characteristics inherent to the anatomical locales where EGC manifests. Intriguingly, severe submucosal fibrosis and ulceration emerged as prevalent phenomena within the gastric angulus. It’s known that in China, gastric ulcers are commonly found in the gastric angulus, and prior literature has suggested a correlation between severe submucosal fibrosis and ulceration [11, 24, 25]. Oi et al. have proposed the double-regulation theory to explain why ulcers are more often observed in the gastric angulus and why ulcers tend to recur in the same or adjacent sites [26]. The recurrence of ulcers may be attributed to the presence of scars and submucosal fibrosis, which potentially suggests that submucosal fibrosis could similarly prevail within the gastric angulus. Multiple factors may be related to the increased severity of submucosal fibrosis in the gastric angulus. Anatomically, the angulus is subjected to significant mechanical stress and stasis, resulting in mucosal injury and subsequent fibrotic healing [27]. Chronic ulceration in this region often leads to structural changes and scarring, potentially making the gastric angulus susceptible to ulcer recurrence and fibrosis [28]. Persistent inflammation, driven by macrophages and neutrophils, may further exacerbate mucosal damage and fibrosis [28, 29]. Moreover, H. pylori infection may contributes to ulcer recurrence and fibrosis by and promoting epithelial-mesenchymal transition (EMT) through the activation of gastric fibroblasts [30]. Other underlying mechanisms, such as genetic factors with predictive values that exacerbate fibrosis, are expected to be elucidated in the future.

The discovery of significant submucosal fibrosis at the gastric angulus has introduced additional complexity to endoscopic maneuvers in this region. Our findings align with a recent report that underscores severe submucosal fibrosis as a notable risk factor for micro-perforation during ESD. When fibrosis is detected beneath the lesion, the insufficient submucosal cushion caused by unsuccessful lifting and the firm fibrotic tissue are likely to induce surgical mishaps owing to improper knife positioning. While patients remain susceptible to micro-perforation risks, timely closure of the perforation using endoclips can obviate the need for surgical intervention. Swift sealing of the aperture effectively minimizes any leakage of gastric contents and reduces the need for nasogastric drainage [31]. Furthermore, the severe submucosal fibrosis at the angulus often leads to an ambiguous submucosal tissue plane and frequent failures when attempting to elevate the lesion through injections [32]. Consequently, these factors contribute to an increased rate of non-*en bloc* resection and perforation complications, and prolonged procedure duration, as observed in our study as well.

Overcoming the obstacles of dissecting fibrotic submucosal tissue at the angulus while achieving higher rates of *en bloc* resection and shorter procedure duration remains a formidable challenge. In such circumstances, traction methods and flexible endoscopes become viable options. For example, the implementation of traction-assisted endoscopic submucosal dissection (TA-ESD), which employs dental floss and a clip, has been documented as a valuable approach to shorten procedure duration and reduce the risk of intraoperative perforation, even for lesions in the lesser curvature [33, 34]. Similarly, Kitamura employed the pocket-creation method to facilitate gastric ESD along the lesser curvature of the angulus, which enabled precise horizontal and straight dissection by effectively stabilizing the endoscope’s tip within the pocket and reducing stomach insufflation [10]. Therefore, endoscopists should consider appropriate techniques, the extent of fibrosis, and their proficiency when performing ESD at angulus to avoid prolonged procedure times and partial resections. Also, the efficacy and safety of these new techniques for treating angulus EGC are encouraged to be further validated in the future.

Further subgroup analysis of the clinicopathological characteristics of EGC at the gastric angulus revealed that, within the mucosal lesion invasion and differentiated histology subgroups, perforation and procedure time were independent risk factors for severe fibrosis following ESD. In contrast, submucosal invasion and undifferentiated histology were not significant factors influencing the treatment outcomes of severe fibrosis in gastric angulus EGC, warranting further investigation. Additionally, a median follow-up of 68 months for gastric angulus EGC patients showed no significant difference in DFS and OS between the F2 group and the F0 + F1 group. This suggests that the severity of fibrosis in EGC at the angulus does not affect long-term prognosis but impacts only short-term ESD outcomes, specifically perforation complication and procedure time.

In all, our research may have a significant impact on the clinical practice of ESD for angulus EGC. Preoperative assessment is vital, with ESD practitioners using advanced techniques like high-resolution endoscopy, narrow-band imaging (NBI), and ultrasonography (EUS) for accurate ESD planning [35, 36]. Adjustments in surgical strategy, such as traction-assisted techniques or flexible endoscopy, are recommended to address fibrosis-related challenges and reduce complications. Improved intraoperative management, especially for prolonged surgeries, is necessary to minimize risks of bleeding and perforation. Enhancing postoperative care includes monitoring patient recovery, timely use of PPIs and antibiotics, and necessary follow-up examinations. Our findings may further necessitate updates to clinical guidelines and the development of new risk stratification tools for angulus EGC, highlighting the importance of increased awareness among ESD practitioners.

The present study has its limitations. Firstly, the present study, being retrospective, inherently faced potential selection bias and data integrity issues; we used stringent inclusion criteria and rigorously checked data completeness to mitigate this. Secondly, due to current constraints such as limited time and energy, this study was conducted at a single center. Future research may involve broader collaborations and multi-center designs to improve validation efforts. Additionally, this study specifically examined data from EGC patients who underwent ESD, excluding those who underwent surgical resection or remained untreated. Despite these limitations, it was meaningful to study the characteristics of EGC at different anatomical sites, and the discovery of severe submucosal fibrosis at the gastric angulus is a valuable finding of this study. Future studies may delve into unveiling predictive factors that contribute to the development of severe submucosal fibrosis at the gastric angulus or exploring new therapeutic approaches.

## Conclusion

The gastric angulus, a remarkably diminutive area, emerged as the second-most prevalent site for EGCs patients. Additionally, severe submucosal fibrosis and ulceration were frequently observed in this area, leading to increased perforation risks and prolonged procedure duration. Consequently, endoscopists ought to proficiently manage lesions at these sites during dissection in EGC patients.

### Electronic supplementary material

Below is the link to the electronic supplementary material.

Supplementary Material 1

## Data Availability

Data used in this study are available from the corresponding author upon reasonable request.
