# Supplementary material for: Unveiling clinicopathologic features and outcomes for endoscopic submucosal dissection of early gastric cancer at gastric angulus in China
Source: BMC Cancer. 2024 Jul 30;24:924. doi: 10.1186/s12885-024-12610-1 (PMC11290107; doi:10.1186/s12885-024-12610-1)
Supplement: Supplementary file 1 — Supplementary Material 1 [file 12885_2024_12610_MOESM1_ESM.docx]

**Supporting information**

**Unveiling Clinicopathologic Features and Outcomes for Endoscopic Submucosal Dissection of Early Gastric Cancer at Gastric Angulus In China**

Qiaoyan Wu^1, 2‡^, Tongyu Li^3‡^, Yangyang Cui^4^, Haizhong Jiang^1, 2^, Yangbo Fu^1, 2^, Qi Jiang^1, 2^, Xiaoyun Ding^1, 2*^

^1^Department of Gastroenterology, The First Affiliated Hospital of Ningbo University, No. 59, Liuting Street, Ningbo 315010, Zhejiang Province, China

^2^Ningbo Key Laboratory of Translational Medicine Research on Gastroenterology and Hepatology, No. 59, Liuting Street, Ningbo 315010, Zhejiang Province, China
^3^Department of Hematology, The First Affiliated Hospital of Ningbo University, No. 59, Liuting Street, Ningbo 315010, Zhejiang Province, China
^4^Department of Histopathology, Ningbo Diagnostic Pathology Center, No. 685, North Huancheng Road, Ningbo 315021, Zhejiang Province, China

‡These authors contributed equally.

Correspondence:

Xiaoyun Ding, Ph D,

E-mail: [dyyyding@126.com](mailto:dyyyding@126.com)


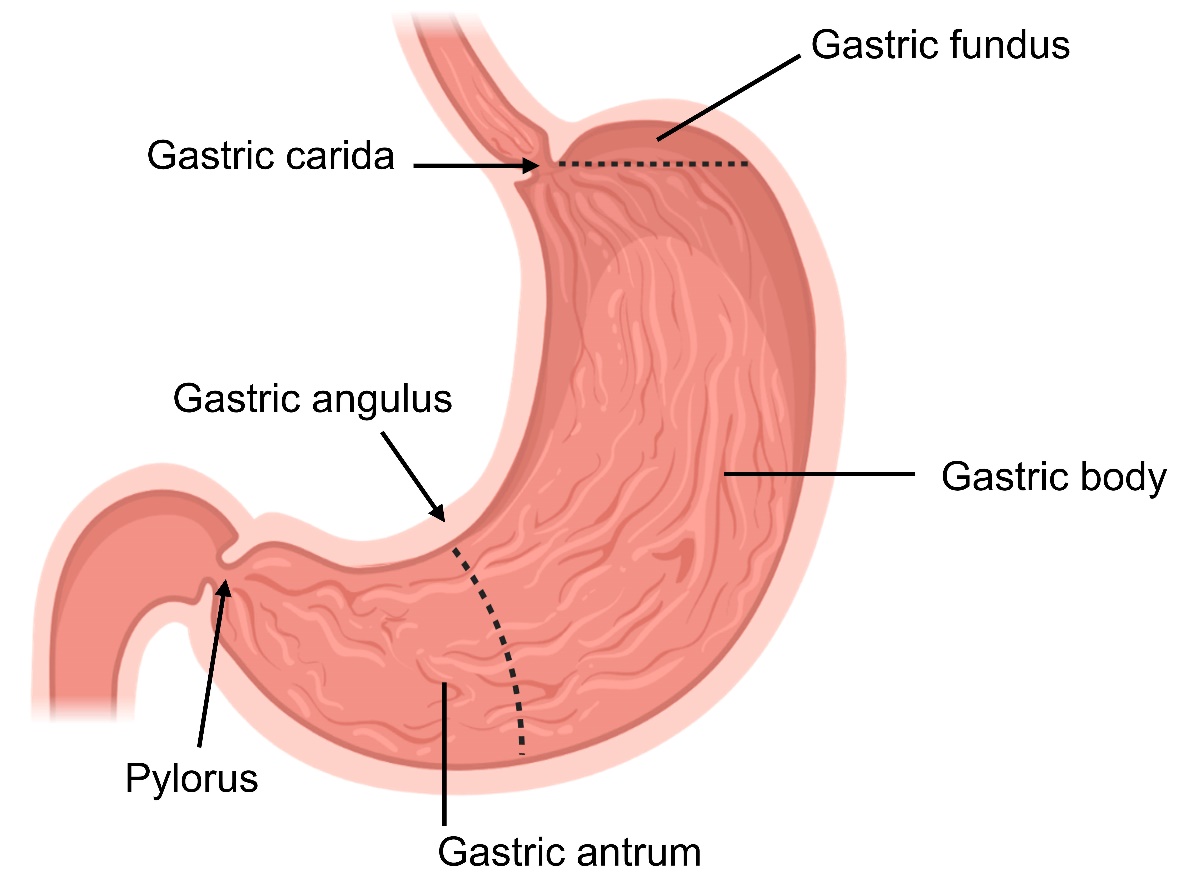


**Figure S1.** Gastric locations of EGC (created with BioRender.com).

**Definitions**

The classification system employed in this study involved three distinct stages of fibrosis during ESD. Two experienced endoscopists jointly determined the endoscopic fibrosis grade. The first stage, F0, was fibrosis-free and blue. A white web-like structure indicated moderate fibrosis in F1 in the submucosal layers of blue. The advanced stage, F2, showed severe fibrosis with a white muscular-like structure in the submucosal layers of white. "*en bloc* resection" refers to a procedure in which tissue is removed in one continuous piece rather than in pieces. An *en bloc* resection with lateral and vertical tumor-negative margins was considered a definition of complete resection. A complete resection without lymphatic infiltration and meeting absolute or expanded indications was referred to as a curative resection. Delayed bleeding, in the context of ESD, refers to the occurrence of gastrointestinal bleeding that is discernible through clinical symptoms and laboratory changes. The diagnosis of perforation was established through the utilization of endoscopic visualization of mesenteric fat or the identification of free air on an abdominal radiograph or computed tomography scan. The duration of the procedure was measured from the moment of the initial mucosal incision to the end of tumor resection.


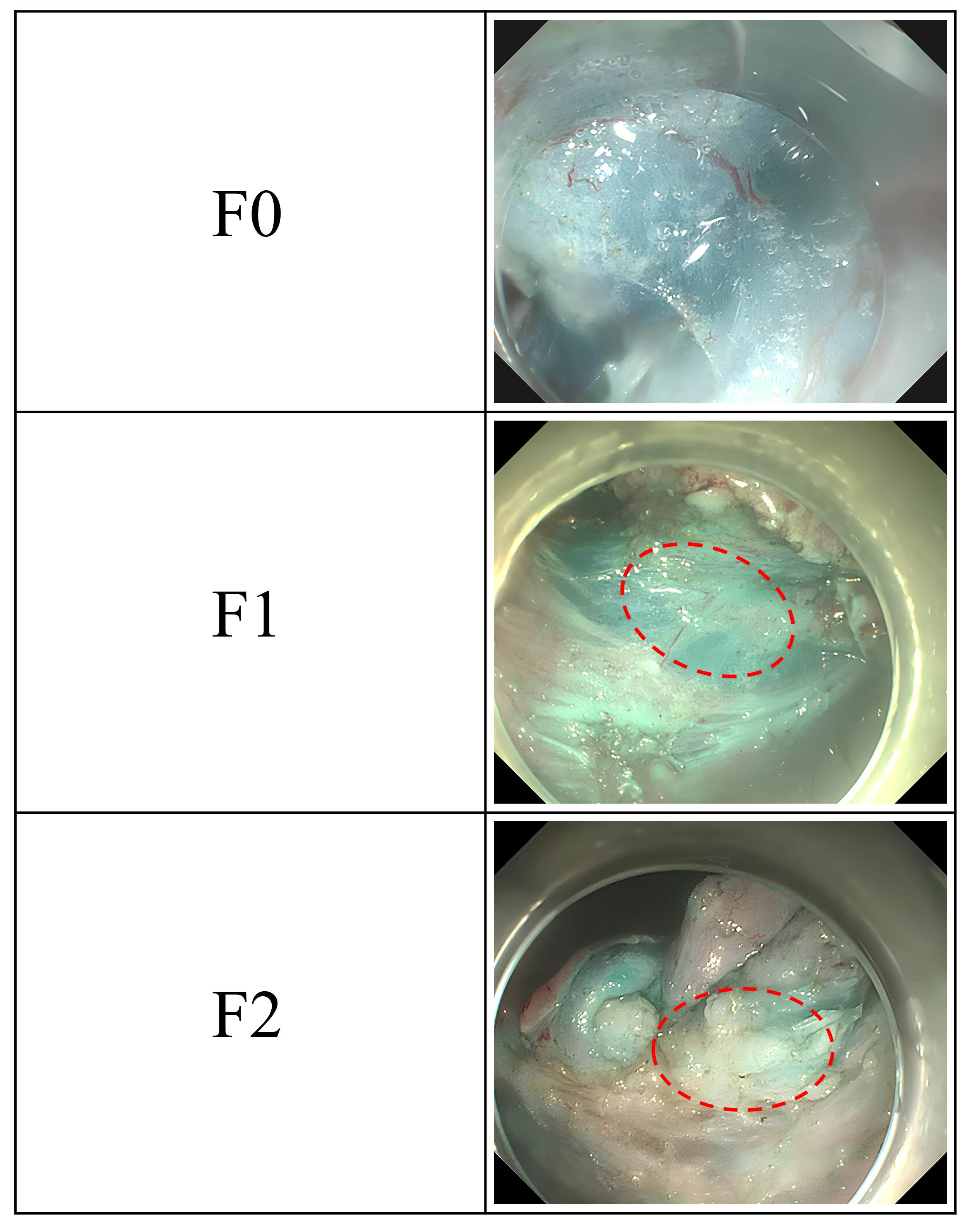


**Figure S2.** The degree of fibrosis present in the submucosal layers. F0 denotes an absence of fibrosis, manifesting as a blue transparent layer; F1 designates mild fibrosis, discernible as a white reticulated structure amidst the blue submucosal layer; and F2 indicates severe fibrosis, visible as a white muscular structure, devoid of the transparent blue layer within the submucosal layer. Specific focal lesions were outlined with dashed red borders


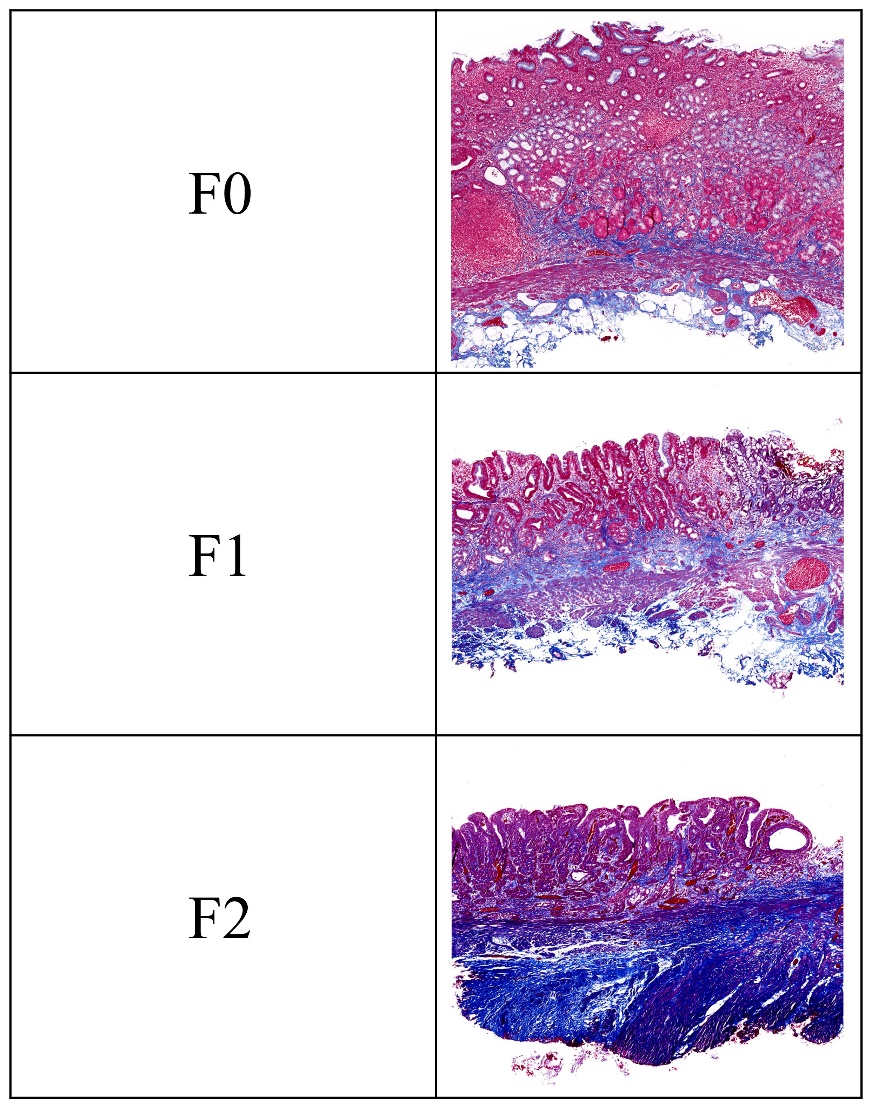


**Figure S3.** The present study showcases endoscopic submucosal dissections with typical observations of submucosal fibrosis. A shows no fibrosis (designated as F0), B indicates mild fibrosis (F1), and C represents severe fibrosis (F2). Masson's trichrome staining technique was employed with an original magnification at 40x.

| **TABLE S1**. Comparison between gastric angulus and gastric body: a univariate analysis | | | | | |
| --- | --- | --- | --- | --- | --- |
| **Variables** | **Body**  **(n = 123)** | **Angulus**  **(n = 161)** | ***P* valve** | ***Cohen’s/Phi*** | |
| Male sex, n (%) | 91 (74.0) | 109 (67.7) | 0.250 | 0.068 | |
| Age, yr | 63.4 (7.4) | 65.3 (9.0) | 0.168 | 0.045 | |
| <65 , n (%) | 71 (57.7) | 80 (49.7) | 0.179 | 0.080 | |
| ≥65 , n (%) | 52 (42.3) | 81 (50.3) |  | |  |
| Comorbidities, n (%) |  |  |  | |  |
| COPD | 7 (5.7) | 3 (1.9) | 0.107 | | 0.103 |
| Diabetes | 11 (8.9) | 7 (4.3) | 0.115 | | 0.093 |
| Hypertension | 44 (35.8) | 45 (28.0) | 0.159 | | 0.084 |
| Tumor size (mm) |  |  | 0.988 | | 0.001 |
| <20 mm, n (%) | 74 (60.2) | 97 (60.2) |  | |  |
| ≥20 mm, n (%) | 49 (39.8) | 64 (39.8) |  | |  |
| Macroscopic type, n (%) |  |  | 0.054 | | 0.114 |
| Elevated | 51 (41.5) | 49 (30.4) |  | |  |
| Flat or depressed | 72 (58.5) | 112 (69.6) |  | |  |
| Submucosal fibrosis, n (%) |  |  | <0.001 | | 0.711 |
| No or mild (F0 + F1) | 116 (94.3) | 131 (81.4) |  | |  |
| Severe (F2) | 7 (5.7) | 30 (18.6) |  | |  |
| Ulceration | 22 (17.5) | 61 (37.9) | <0.001 | | 0.611 |
| Number of tumors, n (%) |  |  | 0.445 | | 0.045 |
| Single | 103 (83.7) | 140 (87.1) |  | |  |
| Multiple | 20 (16.4) | 21 (12.9) |  | |  |
| Depth of invasion, n (%) |  |  | 0.120 | | 0.092 |
| Mucosal lesion | 110 (89.4) | 152 (94.4) |  | |  |
| Submucosal invasion | 13 (10.6) | 9 (5.6) |  | |  |
| Resection margin involvement, n (%) |  |  |  | |  |
| Lateral margin (+) | 0 (0.0) | 3 (1.9) | 0.349 | | 0.090 |
| Vertical margin (+) | 2 (1.6) | 3 (1.9) | 1.000 | | 0.009 |
| Lymphatic invasion, n (%) | 2 (1.6) | 5 (3.1) | 0.681 | | 0.047 |
| Histology, n (%) |  |  | 0.348 | | 0.056 |
| Differentiated | 114 (92.7) | 144 (89.4) |  | |  |
| Undifferentiated | 9 (7.3) | 17 (10.6) |  | |  |
| *En bloc* resection, n (%) | 121 (98.4) | 149 (92.5) | 0.025 | | 0.322 |
| Complete resection, n (%) | 119 (96.7) | 146 (90.7) | 0.043 | | 0.211 |
| Curative resection, n (%) | 118 (95.9) | 144 (89.4) | 0.043 | | 0.211 |
| Complications, n (%) |  |  |  | |  |
| Perforation | 2 (1.6) | 11 (6.8) | 0.038 | | 0.312 |
| Delayed bleeding | 4 (3.3) | 8 (5.0) | 0.476 | | 0.042 |
| Additional gastrectomy, n (%) | 5 (4.1) | 7 (4.3) | 0.907 | | 0.007 |
| Lymph node metastasis, n (%) | 2 (1.6) | 1 (0.6) | 0.814 | | 0.049 |
| Procedure time (min) | 75. 0 (50.0) | 83.0 (40.0) | 0.039 | | 0.055 |
| Hospital stay (day) | 9.0 (3.0) | 9.0 (2.0) | 0.434 | | 0.009 |

| **TABLE S2**. Detailed comparison of the following variables in the gastric body and angulus | | | | | |
| --- | --- | --- | --- | --- | --- |
|  | **Variables** | **Anterior** | **Posterior** | **Lesser curvature** | **Greater curvature** |
| **Body** | F0 + F1 | 18 (100.0) | 27 (93.1) | 49 (94.2) | 22 (91.7) |
|  | F2 | 0 (0.0) | 2 (6.9) | 3 (5.8) | 2 (8.3) |
|  | Total | 18 | 29 | 52 | 24 |
| **Angulus** | F0 + F1 | 22 (100.0) | 51 (87.9) | 21 (75.0) | 37 (69.8) |
|  | F2 | 0 (0.0) | 7 (12.1) | 7 (25.0) | 16 (30.2) |
|  | Total | 22 | 58 | 28 | 53 |
|  | ***P* valve** | - | 0.709 | 0.033 | 0.036 |
|  | ***Phi*** |  | 0.080 | 0.327 | 0.329 |
| **Body** | Ulceration | 3 (16.7) | 7 (24.1) | 10 (19.2) | 2 (8.3) |
| **Angulus** | Ulceration | 0 (0.0) | 18 (31.0) | 18 (64.3) | 25 (47.2) |
|  | ***P* valve** | 0.083 | 0.503 | <0.001 | 0.001 |
|  | ***Phi*** | 0.203 | 0.062 | 0.577 | 0.542 |


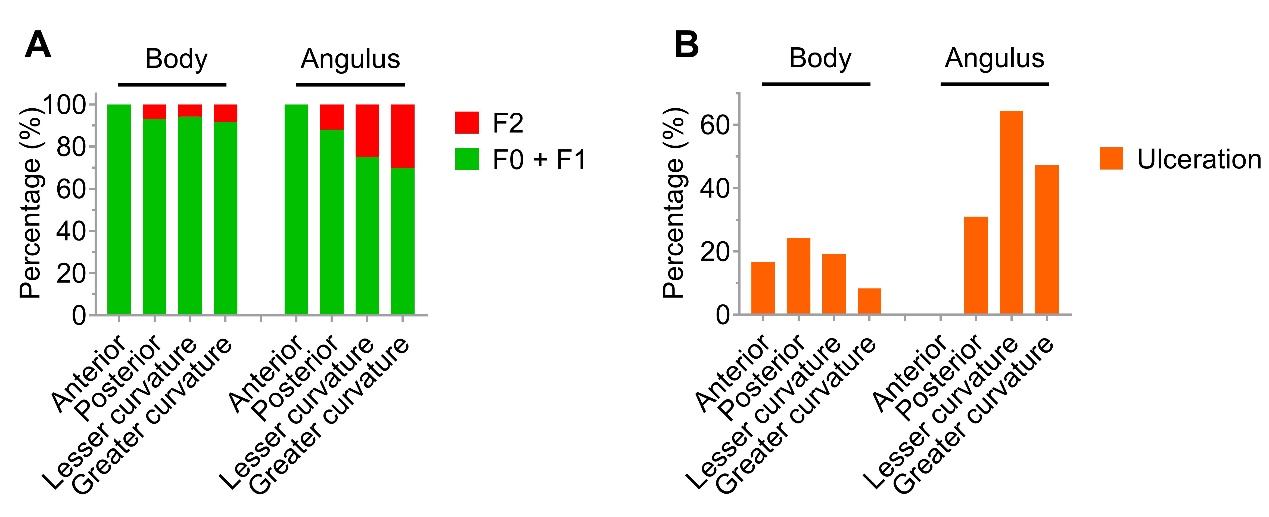


**Figure S4.** Bar graph showing the incidence of severe submucosal fibrosis (**A**) and ulceration (**B**) in the anterior, posterior, lesser curvature, and greater curvature of the gastric body and angulus.

| **TABLE S3.** Logistic regression analysis of risk factor for severe submucosal fibrosis in different stratification | | | | | | |  |  |  |  |  |  |
| --- | --- | --- | --- | --- | --- | --- | --- | --- | --- | --- | --- | --- |
| **Variables** | **F0 + F1**  **(n = 131)** | | **F2**  **(n = 30)** | **UOR***  **(95% CI)** | ***P* valve** | **AOR***  **(95% CI)** | ***P* valve** |  |  |  |  |  |
| **Mucosal lesion invasion, n (%)** | 128 (97.7) | | 24 (80.0) |  |  |  |  |  |  |  |  |  |
| Male sex, n (%) | 81 (63.3) | | 20 (83.3) | 2.901 (0.935-9.000) | 0.065 |  |  |  |  |  |  |  |
| Age, yr | 64.5 (9.0) | | 71.2 (7.8) | 1.124 (0.097-1.301) | 0.114 |  |  |  |  |  |  |  |
| Tumor size (mm) |  | |  | 3.413 (1.381-8.436) | 0.008 | 2.026 (0.568-7.223) | 0.276 |  |  |  |  |  |
| < 20 mm, n (%) | 86 (67.2) | | 9 (37.5) |  |  |  |  |  |  |  |  |  |
| ≥ 20 mm, n (%) | 42 (32.8) | | 15 (62.5) |  |  |  |  |  |  |  |  |  |
| Macroscopic type, n (%) |  | |  | 0.569 (0.232-1.396) | 0.215 |  |  |  |  |  |  |  |
| Elevated | 37 (28.9) | | 10 (41.7) |  |  |  |  |  |  |  |  |  |
| Flat or depressed | 91 (71.1) | | 14 (58.3) |  |  |  |  |  |  |  |  |  |
| Ulceration, n (%) | 35 (27.3) | | 18 (75.0) | 7.971 (2.926-21.721) | <0.001 | 2.957 (0.841-10.391) | 0.091 |  |  |  |  |  |
| Number of tumors, n (%) |  | |  | 0.522 (0.113-2.402) | 0.404 |  |  |  |  |  |  |  |
| Single | 109 (85.2) | | 22 (91.7) |  |  |  |  |  |  |  |  |  |
| Multiple | 19 (14.9) | | 2 (8.3) |  |  |  |  |  |  |  |  |  |
| Resection margin involvement, n (%) | | | |  |  |  |  |  |  |  |  |  |
| Lateral margin (+) | 2 (1.6) | | 1 (4.2) | 2.739 (0.238-31.465) | 0.419 |  |  |  |  |  |  |  |
| Vertical margin (+) | 0 (0.0) | | 1 (4.2) | - | 1.000 |  |  |  |  |  |  |  |
| Lymphatic invasion, n (%) | 4 (3.1) | | 1 (4.2) | 1.348 (0.144-12.610) | 0.794 |  |  |  |  |  |  |  |
| Histology, n (%) |  | |  | 5.000 (1.554-16.089) | 0.007 | 0.548 (0.055-5.517) | 0.610 |  |  |  |  |  |
| Differentiated | 120 (93.8) | | 18 (75.0) |  |  |  |  |  |  |  |  |  |
| Undifferentiated | 8 (6.3) | | 6 (25.0) |  |  |  |  |  |  |  |  |  |
| Complications, n (%) | | | | | | |  |  |  |  |  |  |
| Perforation | 2 (1.6) | | 7 (29.2) | 25.941(4.977-135.217) | <0.001 | 18.475 (2.055-166.109) | 0.009 |  |  |  |  |  |
| Delayed bleeding | 5 (3.9) | | 3 (12.5) | 3.514 (0.781-15.818) | 0.218 |  |  |  |  |  |  |  |
| Additional gastrectomy, n (%) | 2 (1.6) | | 3 (12.5) | 9.000 (1.418-57.117) | 0.020 | 2.695 (0.119-60.804) | 0.533 |  |  |  |  |  |
| Lymph node metastasis, n (%) | 0 (0.0) | | 1 (4.2) | - | 1.000 |  |  |  |  |  |  |  |
| Procedure time (min) | 75 (22.7) | | 110 (31.1) | 1.051 (1.030-1.072) | <0.001 | 1.034 (1.010-1.059) | 0.006 |  |  |  |  |  |
| Hospital stay (day) | 9 (2.1) | | 10.0 (2.3) | 1.216 (1.018-1.452) | 0.031 | 1.127(0.875-1.451) | 0.355 |  |  |  |  |  |
| **Submucosal invasion, n (%)** | 3 (2.3) | | 6 (20.0) |  |  |  |  |  |  |  |  |  |
| Male sex, n (%) | 3 (100.0) | | 5 (83.3) | - | 1.000 |  |  |  |  |  |  |  |
| Age, yr | 69.5 (0.7) | | 68.7 (5.5) | 0.935 (0.562-1.555) | 0.796 |  |  |  |  |  |  |  |
| Tumor size (mm) |  | |  | 2.500 (0.100-62.605) | 0.577 |  |  |  |  |  |  |  |
| < 20 mm, n (%) | 1 (33.3) | | 1 (16.7) |  |  |  |  |  |  |  |  |  |
| ≥ 20 mm, n (%) | 2 (66.7) | | 5 (83.3) |  |  |  |  |  |  |  |  |  |
| Macroscopic type, n (%) |  | |  | - | 0.999 |  |  |  |  |  |  |  |
| Elevated | 0 (0.0) | | 2(33.3) |  |  |  |  |  |  |  |  |  |
| Flat or depressed | 3 (100.0) | | 4 (66.7) |  |  |  |  |  |  |  |  |  |
| Ulceration | 2 (66.7) | | 6 (100.0) | - | 1.000 |  |  |  |  |  |  |  |
| Number of tumors, n (%) |  | |  | - | 0.327 |  |  |  |  |  |  |  |
| Single | 3 (100.0) | | 6 (100.0) |  |  |  |  |  |  |  |  |  |
| Multiple | 0 (0.0) | | 0 (0.0) |  |  |  |  |  |  |  |  |  |
| Resection margin involvement, n (%) | | |  |  |  |  |  |  |  |  |  |  |
| Lateral margin (+) | 0 (0.0) | | 0 (0.0) | - | 0.327 |  |  |  |  |  |  |  |
| Vertical margin (+) | 0 (0.0) | | 2 (33.3) | - | 0.999 |  |  |  |  |  |  |  |
| Lymphatic invasion, n (%) | 0 (0.0) | | 0 (0.0) | - | 0.327 |  |  |  |  |  |  |  |
| Histology, n (%) |  | |  | 1.000 (0.053-18.912) | 1.000 |  |  |  |  |  |  |  |
| Differentiated | 2 (66.7) | | 4 (66.7) |  |  |  |  |  |  |  |  |  |
| Undifferentiated | 1 (33.3) | | 2 (33.3) |  |  |  |  |  |  |  |  |  |
| Complications, n (%) |  | |  |  |  |  |  |  |  |  |  |  |
| Perforation | 0 (0.0) | | 2 (33.3) | - | 0.999 |  |  |  |  |  |  |  |
| Delayed bleeding | 0 (0.0) | | 0 (0.0) | - | 0.327 |  |  |  |  |  |  |  |
| Additional gastrectomy, n (%) | 1 (33.3) | | 1 (16.7) | 0.400 (0.016-10.017) | 0.577 |  |  |  |  |  |  |  |
| Lymph node metastasis, n (%) | 0 (0.0) | | 0 (0.0) | - | 0.327 |  |  |  |  |  |  |  |
| Procedure time (min) | 61.7 (20.2) | | 111.6 (29.9) | 1.076 (0.981-1.180) | 0.119 |  |  |  |  |  |  |  |
| Hospital stay (day) | 10.0 (1.0) | | 9.2 (2.6) | 0.817 (0.411-1.627) | 0.566 |  |  |  |  |  |  |  |
| **Differentiated Histology, n (%)** | 122 (93.1) | | 22 (73.3) |  |  |  |  |  |  |  |  |  |
| Male sex, n (%) | 79 (64.8) | | 19 (86.4) | 3.447 (0.965-12.313) | 0.057 |  |  |  |  |  |  |  |
| Age, yr | 65.3 (7.6) | | 72.0 (7.0) | 1.151 (0.993-1.336) | 0.062 |  |  |  |  |  |  |  |
| Tumor size (mm) |  | |  | 3.868 (1.497-9.997) | 0.005 | 2.039 (0.601-6.918) | 0.253 |  |  |  |  |  |
| < 20 mm, n (%) | 84 (68.9) | | 8 (36.4) |  |  |  |  |  |  |  |  |  |
| ≥ 20 mm, n (%) | 38 (31.1) | | 14 (63.6) |  |  |  |  |  |  |  |  |  |
| Macroscopic type, n (%) |  | |  | 0.605 (0.237-1.540) | 0.290 |  |  |  |  |  |  |  |
| Elevated | 36 (29.5) | | 9 (40.9) |  |  |  |  |  |  |  |  |  |
| Flat or depressed | 86 (70.5) | | 13 (59.1) |  |  |  |  |  |  |  |  |  |
| Depth of invasion, n (%) |  | |  | 13.333(2.275-78.137) | 0.004 | 9.609(0.929-89.733) | 0.051 |  |  |  |  |  |
| Mucosal lesion | 120 (98.4) | | 18 (81.8) |  |  |  |  |  |  |  |  |  |
| Submucosal invasion | 2 (1.6) | | 4 (18.2) |  |  |  |  |  |  |  |  |  |
| Ulceration | 32 (26.2) | | 16 (72.7) | 7.500 (2.701-20.826) | <0.001 | 3.099 (0.882-10.894) | 0.078 |  |  |  |  |  |
| Number of tumors, n (%) |  | |  | 0.542 (0.117-2.513) | 0.434 |  |  |  |  |  |  |  |
| Single | 103 (84.4) | | 20 (90.0) |  |  |  |  |  |  |  |  |  |
| Multiple | 19 (15.6) | | 2 (9.1) |  |  |  |  |  |  |  |  |  |
| Resection margin involvement, n (%) | | |  |  |  |  |  |  |  |  |  |  |
| Lateral margin (+) | 2 (1.6) | | 1 (4.5) | 2.857 (0.248-32.938) | 0.400 |  |  |  |  |  |  |  |
| Vertical margin (+) | 0 (0.0) | | 0 (0.0) | - |  |  |  |  |  |  |  |  |
| Lymphatic invasion, n (%) | 4 (3.3) | | 1 (4.5) | 1.405 (0.150-13.195) | 0.766 |  |  |  |  |  |  |  |
| Complications, n (%) |  | |  |  |  |  |  |  |  |  |  |  |
| Perforation | 2 (1.6) | | 5 (22.7) | 17.647 (3.170-98.224) | 0.001 | 11.410 (1.133-114.924) | 0.039 |  |  |  |  |  |
| Delayed bleeding | 4 (3.3) | | 2 (9.1) | 2.950 (0.506-17.186) | 0.229 |  |  |  |  |  |  |  |
| Additional gastrectomy, n (%) | 0 (0.0) | | 0 (0.0) | - | - |  |  |  |  |  |  |  |
| Lymph node metastasis, n (%) | 0 (0.0) | | 1 (4.5) | - | 1.000 |  |  |  |  |  |  |  |
| Procedure time (min) | 73.9 (22.0) | | 100.0 (26.5) | 1.046 (1.024-1.068) | <0.001 | 1.029 (1.004-1.055) | 0.022 |  |  |  |  |  |
| Hospital stay (day) | 8.9 (2.1) | | 9.8 (2.2) | 1.178 (0.980-1.416) | 0.081 |  |  |  |  |  |  |  |
| **Undifferentiated Histology, n (%)** | | 9 (6.9) | 8 (2.7) |  |  |  |  |  |  |  |  |  |
| Male sex, n (%) | 5 (55.6) | | 6 (75.0) | 2.400 (0.303-19.041) | 0.407 |  |  |  |  |  |  |  |
| Age, yr | 58.3 (14.8) | | 65.0 (5.0) | 1.066 (0.876-1.296) | 0.524 |  |  |  |  |  |  |  |
| Tumor size (mm) |  | |  | 1.500 (0.181-12.459) | 0.707 |  |  |  |  |  |  |  |
| < 20 mm, n (%) | 3 (33.3) | | 2 (25.0) |  |  |  |  |  |  |  |  |  |
| ≥ 20 mm, n (%) | 6 (66.7) | | 6 (75.0) |  |  |  |  |  |  |  |  |  |
| Macroscopic type, n (%) |  | |  | 0.208 (0.017-2.600) | 0.233 |  |  |  |  |  |  |  |
| Elevated | 1 (11.1) | | 3 (37.5) |  |  |  |  |  |  |  |  |  |
| Flat or depressed | 8 (88.9) | | 5 (62.5) |  |  |  |  |  |  |  |  |  |
| Depth of invasion, n (%) |  | |  | 2.667 (0.193-36.756) | 0.464 |  |  |  |  |  |  |  |
| Mucosal lesion | 8 (88.9) | | 6 (75.0) |  |  |  |  |  |  |  |  |  |
| Submucosal invasion | 1 (11.1) | | 2 (25.0) |  |  |  |  |  |  |  |  |  |
| Ulceration | 5 (55.6) | | 8 (100.0) | - | 0.999 |  |  |  |  |  |  |  |
| Number of tumors, n (%) |  | |  | 1.178 (0.980-1.416) | 0.081 |  |  |  |  |  |  |  |
| Single | 9 (100.0) | | 8 (100.0) | - | 0.808 |  |  |  |  |  |  |  |
| Multiple | 0 (0.0) | | 0 (0.0) |  |  |  |  |  |  |  |  |  |
| Resection margin involvement, n (%) | | |  |  |  |  |  |  |  |  |  |  |
| Lateral margin (+) | 0 (0.0) | | 0 (0.0) | - | 0.808 |  |  |  |  |  |  |  |
| Vertical margin (+) | 0 (0.0) | | 3 (37.5) | - | 0.999 |  |  |  |  |  |  |  |
| Lymphatic invasion, n (%) | 0 (0.0) | | 0 (0.0) | - | 0.808 |  |  |  |  |  |  |  |
| Complications, n (%) |  | |  |  |  |  |  |  |  |  |  |  |
| Perforation | 0 (0.0) | | 4 (50.0) | - | 0.999 |  |  |  |  |  |  |  |
| Delayed bleeding | 1 (11.1) | | 1 (12.5) | 1.143 (0.060-21.870) | 0.929 |  |  |  |  |  |  |  |
| Additional gastrectomy, n (%) | 3 (33.3) | | 4 (50.0) | 2.000(0.282-14.198) | 0.488 |  |  |  |  |  |  |  |
| Lymph node metastasis, n (%) | 0 (0.0) | | 0 (0.0) | - | 0.808 |  |  |  |  |  |  |  |
| Procedure time (min) | 83.3 (30.8) | | 140.0 (19.3) | 1.089 (1.004-1.183) | 0.041 |  |  |  |  |  |  |  |
| Hospital stay (day) | 9.2 (1.5) | | 9.9 (2.9) | 1.155 (0.731-1.825) | 0.536 |  |  |  |  |  |  |  |

*UOR, Unadjusted OR; AOR, adjusted OR.


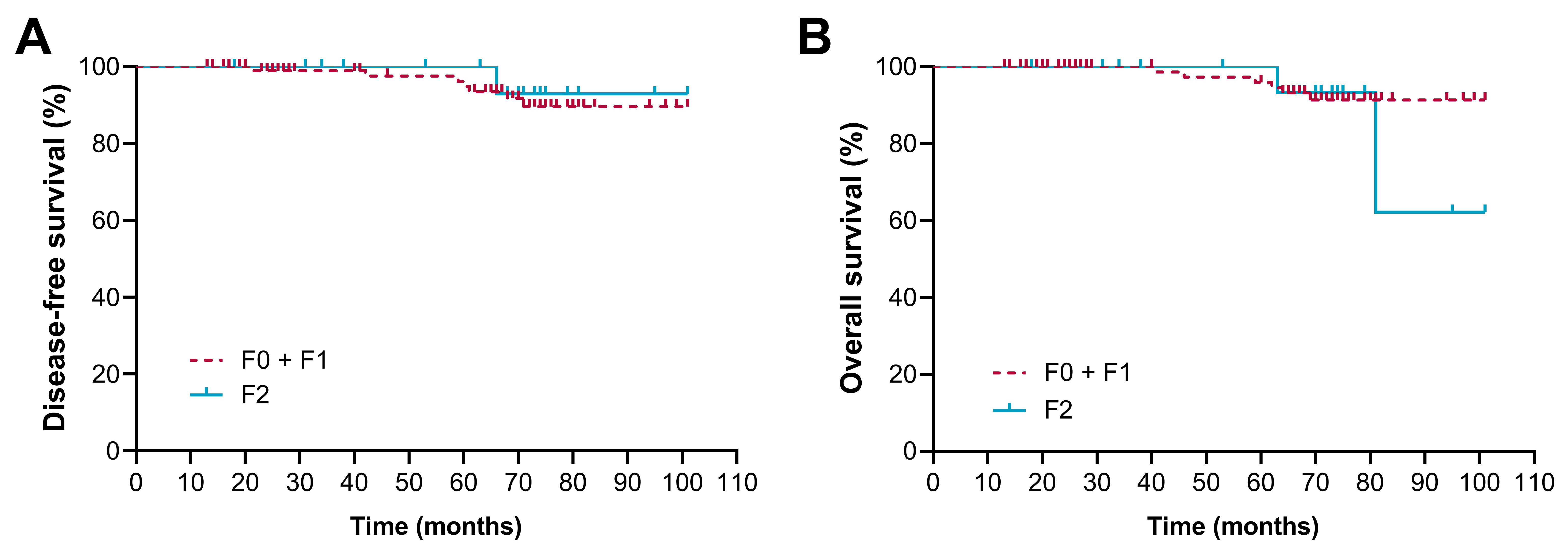


**Figure S5.** Kaplan-Meier analysis for disease-free survival (**A**) and overall survival (**B**) between the F0 + F1 and F2 groups.

**Study Highlights**

**What is know**

With the rapid advancement of endoscopic instruments and technologies, the discernment rates of EGC have witnessed a notable surge in South Korea and Japan. Consequently, this has led to a significant reduction in mortality rates and a marked improvement in the 5-year survival rate, underscoring the favorable outcomes achieved by these nations.

**What is new here**

The gastric angulus ranks as the second most common site of EGCs resectable by ESD. Remarkably, this location displays a heightened prevalence of severe submucosal fibrosis, surpassing other regions within the stomach. Hence, managing patients with EGC at the gastric angulus necessitates meticulous and cautious dissection, as they are prone to be coupled with severe submucosal fibrosis.
